# Expounding the role of tick in Africa swine fever virus transmission and seeking effective prevention measures: A review

**DOI:** 10.3389/fimmu.2022.1093599

**Published:** 2022-12-16

**Authors:** Tianbao Lv, Xufeng Xie, Ning Song, Shilei Zhang, Yue Ding, Kun Liu, Luteng Diao, Xi Chen, Shuang Jiang, Tiger Li, Wenlong Zhang, Yongguo Cao

**Affiliations:** ^1^ Department of Clinical Veterinary Medicine, College of Veterinary Medicine, Jilin University, Changchun, China; ^2^ Portsmouth Abbey School, Portsmouth, RI, United States; ^3^ Key Laboratory for Zoonosis Research, Ministry of Education, College of Veterinary Medicine, Jilin University, Changchun, China

**Keywords:** African swine fever, *Ornithodoros* soft ticks, transmission, anti-tick vaccine, ASFV-G-ΔI177L, prevention

## Abstract

African swine fever (ASF), a highly contagious, deadly infectious disease, has caused huge economic losses to animal husbandry with a 100% mortality rate of the most acute and acute infection, which is listed as a legally reported animal disease by the World Organization for Animal Health (OIE). African swine fever virus (ASFV) is the causative agent of ASF, which is the only member of the Asfarviridae family. *Ornithodoros* soft ticks play an important role in ASFV transmission by active biological or mechanical transmission or by passive transport or ingestion, particularly in Africa, Europe, and the United States. First, this review summarized recent reports on (1) tick species capable of transmitting ASFV, (2) the importance of ticks in the transmission and epidemiological cycle of ASFV, and (3) the ASFV strains of tick transmission, to provide a detailed description of tick-borne ASFV. Second, the dynamics of tick infection with ASFV and the tick-induced immune suppression were further elaborated to explain how ticks spread ASFV. Third, the development of the anti-tick vaccine was summarized, and the prospect of the anti-tick vaccine was recapitulated. Then, the marked attenuated vaccine, ASFV-G-ΔI177L, was compared with those of the anti-tick vaccine to represent potential therapeutic or strategies to combat ASF.

## Introduction

African swine fever (ASF) is a very serious contagious disease prevalent in pigs. The clinical symptoms of African swine fever virus (ASFV)-infected pigs include high fever, vomiting, diarrhea, skin hemorrhages, cyanosis, and abortion in pregnant sows, resulting in a mortality rate of up to 100% in acute ASF (1). Subacute and chronic forms emerge as respiratory signs, intermittent fever, chronic skin ulcers, and arthritis, which are caused by lower virulent strains and exhibit lower mortality rates. ASF infection in domestic pigs causes significant direct economic losses and associated indirect economic losses, such as trade restrictions. Since it was tested in Liaoning province in 2018, ASFV has spread to all mainland provinces in China, causing local pork prices to rise (2, 3) and global pork supply to be affected (4).

ASFV, the only known DNA arbovirus, belongs to the family Asfarviridae. ASFV is extremely stable and spreads easily through the infected swine, contamination during the trade of pig products, or blood feeding of infected *Ornithodoros* vector.

In the first report on ASF, Montgomery et al. speculated that the transmission of ASFV might be related to arthropod vectors, since the virus was not transmitted by contacting between warthogs and domestic pigs. Still, many outbreaks on Kenyan farms could only be attributed to warthogs, as the farms are isolated and no pigs or their products were introduced. However, the biological arthropod vectors of ASFV were not published in Spain until 1962, when Sanchez Botija (1963) confirmed that *Ornithodoros erraticus* could maintain and transmit ASFV (5). Following an investigation by researchers in East Africa, they demonstrated ASFV infection in *Ornithodoros moubata* complex in warthog-occupied animal burrows in Tanzania (6). Plowright et al. demonstrated ASFV proliferation, transstadial transmission, and transovarial transmission in the ticks and sexual transmission, showing that ticks could be competent vectors of the ASFV (7–10). Based on the common characteristics of Iridoviridae with ASFV, Plowright et al. suggested that ASFV may have originated from arthropod vectors and evolved in them (6). A recent molecular study supported this opinion by demonstrating ASFV genome-like segments existing in archived specimens of the *O. moubata* complex. Many studies have shown that ticks were important to the transmission of ASFV, and it might contribute to ASFV evolution (11).

Since *Ornithodoros* play an essential role in ASFV transmission, an anti-tick vaccine could be an alternative to prevent ASF in the absence of commercially available anti-ASFV vaccines. Therefore, a large number of researchers have examined the immune effects of tick components, starting with mixed proteins such as egg vitellin, salivary gland extract, and midgut protein extract, and later with recombinant single proteins. Although there are many kinds of candidate proteins affecting the life cycle of ticks, the ideal anti-tick vaccine antigen has not been screened. It was not until 3 June 2022 that the launch of ASFV-G-ΔI177L became a boon for the pig industry against ASFV. So far, whether anti-tick vaccines prevent ASF needs to be further investigated.

Here, we will summarize the role of ticks in ASFV transmission to evaluate the necessity of anti-tick vaccine development and further compare its advantages and disadvantages with the anti-ASFV vaccine, providing direction and ideas for the prevention of ASF.

## Tick species capable of transmitting ASFV

### Argasidae

It was demonstrated that *Ornithodoros* is the only biological ASFV vector (12). Soft ticks repeatedly take blood meals in both the nymph stage and the adult stage, during which both biological and mechanical transmission of pathogens may be involved. Not all Argasidae can transmit ASFV. According to the epidemiological investigation and laboratory infection research results, it has been confirmed that eight taxa had the ability to transmit ASFV, including *Ornithodoros marocanus*, *Ornithodoros moubata porcinus*, *Ornithodoros puertoricensis*, *O. erraticus*, *Ornithodoros moubata* complex, *Ornithodoros turicata*, *Ornithodoros savignyi*, and *Ornithodoros coriaceus (* 13).

*Ornithodoros erraticus* was the first tick association with ASFV made by Sanchez-Botija and was later recognized as a key factor in maintaining the enzootic cycle of ASFV in the Iberian Peninsula. *Ornithodoros moubata porcinus* was identified to transmit ASFV and successful transmit ASFV 469 days post-infection (dpi) through experimental infection (8). Similarly, experimental infections have demonstrated that other *Ornithodoros* can transmit ASFV, including *O. savignyi* (14), *O. coriaceus (* 15, 16), *O. turicata (* 16), and *O. puertoricensis (* 16, 17). *Ornithodoros marocanus* could transmit ASFV out to 588 days post-infection (dpi), although infected ticks had a 73% mortality (18, 19). ASFV was isolated from *O. moubata* collected in domestic pig sties and houses in certain villages in Mchinji district where there was ASF outbreaks, demonstrating that *O. moubata* can act as a reservoir and potential vector of ASFV (20). Due to transovarial, transstadial, or sexual transmission of ASFV, the tick served as reservoir of ASFV (21).

### Ixodidae

Hard ticks or Ixodidae are one of the most important vectors of veterinary significance in the world. Several species, including *Dermacentor reticulatus* and *Ixodes ricinus*, have been assessed for transmitting ASFV by hard ticks. European hard (ixodid) ticks might be a possible vector of ASFV, as ASFV DNA could be measured for 6 weeks or up to 8 weeks in infected *I. ricinus* or *D. reticulatus*, respectively (22).

Although DNA segments of ASFV were detected in Dermacentor ticks (23), there is no evidence that it can carry and transmit ASFV (24). The investigation showed that the European hard ticks is not a risk factor of biological transmission of ASFV in the Baltic States (25). Although hard ticks cannot be considered a mechanical vector for ASFV because they only take blood once at each stage, ASFV could be detected within a few days of experimental infection and transmitted orally in some tick species (26). The worry is that infected hard ticks can transmit ASFV over long distances because they attached to a host for a long time.

## The role of ticks in the transmission and epidemiological cycle of ASFV

Generally, ASFV transmission occurs through direct contact with infected animals or contaminants or through the bite of a soft tick. Four epidemiological cycles described by Chenais summarize the transmission of ASFV (27). Sylvatic cycle was observed in eastern and southern Africa where the virus was transmitted between warthogs and *O. moubata* complex (28). Tick–pig cycle mainly occurs in sub-Saharan Africa and Iberian Peninsula (29). Transmission during domestic cycle was only linked with domestic pigs, without the presence of infected free-living pigs or tick vector, such as West Africa and Brazil (30, 31). In wild boar–habitat cycle, ASFV transmission depends on carcasses in the habitat (27). This chapter will detail the role of soft ticks in ASFV epidemiological cycles.

### Sylvatic cycle

**Figure 1:**
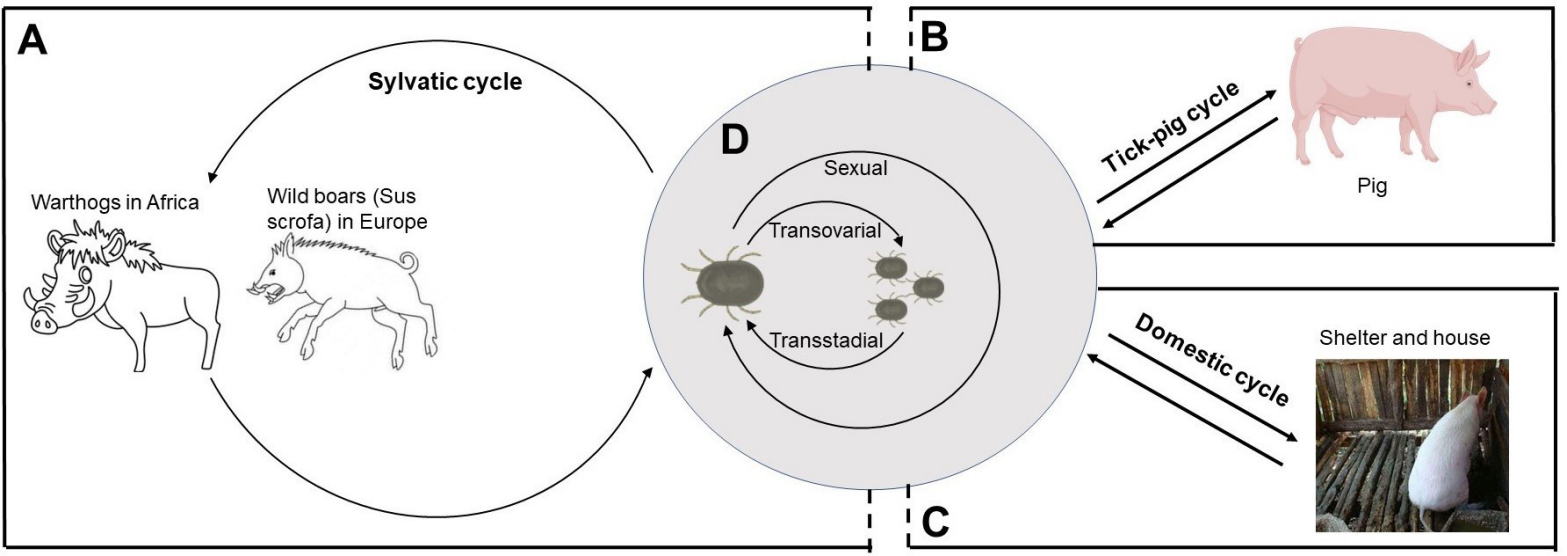
The role of *Ornithodoros* species in ASFV transmission cycles. **(A)** The sylvatic cycle occurs in Africa and Europe where ASFV is transmitted between *Ornithodoros* species and warthogs or wild boars. **(B)** In sub-Saharan Africa and Iberian Peninsula, tick–pig cycle occurs in areas where there is little or no contact between wild and domestic swine. **(C)** In domestic cycle, ASFV-infected ticks usually hide in shelters and houses during the days and have a blood meal at night. **(D)** In *Ornithodoros* species, ASFV is transmitted by transovarial, transstadial, and sexual routes.

Warthogs (*Phacochoerus africanus*), the primary sylvatic host, are asymptomatic but are reservoirs of ASFV. In Africa, the cross-transmission of ASFV between warthogs and soft ticks is a major cause of sylvatic cycle, which is mainly recorded in southern and eastern African countries (**Figure 1**) (32, 33). ASFV transmission occurs between juvenile warthogs and ticks when the ticks were taking blood meals (6, 34). Experimental infected young warthog with ASFV developed viremias between 10^2^ and 10^6^ HAD_50_/ml and high virus concentrations in some lymphatic tissues (higher than or equal to 10^6^ HAD_50_/g) within the first week after infection. The ASFV titer in lymph nodes did not decrease significantly and kept domestic pigs infectious within 33 dpi (35). The sylvatic cycle occurred in eastern and southern Africa where ASFV was transmitted between *O. moubata* complex and warthogs (28). Because the bush pig’s behavior is less conducive to interaction with soft ticks, it is generally thought to play a smaller role in the sylvatic cycle than the warthog, although it can also be infected and transmit ASFV (32, 33, 36, 37).

Similarly, the sylvatic cycle was present in Europe, which involved *O. erraticus* and wild boar (*Sus scrofa*) (**Figure 1**) (33). However, unlike the African system, ASFV may persist in wild and domestic pig populations through horizontal transmission without soft ticks (38).

### Tick–pig cycle

The tick–pig cycle, described mainly in sub-Saharan Africa, is the best evidence that ticks are important risk factors of ASFV transmission (**Figure 1**). In Africa and Madagascar, the isolation of infected *O. moubata* complex in areas with ASF outbreaks suggested that soft ticks were a key risk factor of ASFV maintenance (20, 39, 40). In the Iberian Peninsula, it is also observed that *O. erraticus* were linked with ASFV maintenance (29, 41–43). Among the risk factors for the 2018–2020 ASF outbreak in China reviewed by Cheng et al., swill feeding, live pig transport, and vehicles were listed as the important risk factors (1). Although there is no evidence to link the outbreak of ASF in China to ticks, soft ticks are still classified as a low risk of ASF outbreak (1). In particular, according to the model prediction of Li et al., the southeast coast or central region of China is suitable for ASF distribution, and its environment is suitable for soft ticks, indicating that the tick–pig cycle may promote the outbreak of ASF in China (44).

### Domestic cycle

In the 1980s, there were regular outbreaks of ASF in domestic pigs in Malawi with a low mortality rate, which was called domestic pig–tick cycle since no warthogs or bushpigs in the region were negative for ASFV (20, 45). In shelters of pigs or house of humans, ticks could be found at night but not at day, whose infection rates were comparable with warthog burrows in South Africa, East Africa, and Namibia (**Figure 1**) (20).

## The ASFV strains of tick transmission

ASFV was classified into 8 serogroups and 24 genotypes based on antibody-mediated hemadsorption inhibition and the B646L gene, respectively (46). Here, we summarized the ASFV strains of experimental transmission by the eight ticks mentioned above.

*Ornithodoros marocanus* larvae became infected with ASFV, isolated from an infected domestic pig in Portugal in 1986, by feeding on a viremia pig. Although virus titers decreased with tick development, adult ticks were still able to transmit ASFV to susceptible pigs 588 days later and could recover ASFV 655 days after the infective blood meal (**Table 1**) (19). Experimentally infected *O. puertoricensis* second-instar nymphs with the Dominican Republic isolate (DR-II) of ASFV could transmit virus 239 days after infection (**Table 1**) (17). Hess et al. demonstrated that it was able to transmit ASFV transstadially and transovarially (**Table 1**) (16). *Ornithodoros coriaceus* can be infected with ASFV strain Tengani, Z1, and DR2 together with different mortalities, and infection with the DR2 persisted for 502 days (**Table 1**) (16). *Ornithodoros coriaceus* can pass ASFV transstadially but not transovarially (**Table 1**) (15, 16). *Ornithodoros moubata porcinus-*transmitted ASFV strains include Z1, Uganda, Tengani, Chiredzi/83/1 (Ch1), Pretoriuskop/96/4/1 (Pr4), Crocodile/96/1 (Cr1), and Nooitverwacht/96/6 (No6), with at least 70% of ticks multiplying and persisting for 13–15 months over “Uganda” strain virus (**Table 1**) (8, 16, 47). Sexual transmission, stadial transmission, and ovarial transmission of ASFV were involved in *O. moubata porcinus* (**Table 1**) (10, 16). ASFV strains Tomar 87, OUR T88/1, and ASFV/P99 isolate were able to be transmitted by *O. erraticus*, which harbored ASFV and transmitted it to pigs for at least 588 days after infection (**Table 1**) (43, 48). ASFV strains CHZ T90/1, SIN T90/1, and VIC T90/1, isolated from *O. moubata*, together with Georgia 2007/1 and Ukr12/Zapo isolated from domestic pigs, were able to be transmitted by *O. moubata* (**Table 1**) (37, 49, 50). *Ornithodoros moubata* could transmit Georgia2007/1 and Liv13/33 strains to naive pigs 8 months post-infection (**Table 1**) (50). Only *O. moubata* has been shown to transovarial transmit ASFV (**Table 1**) (51). Infected *O. turicata* transmitted ASFV to susceptible pigs 23 days later (**Table 1**) (16). Infected *O. savignyi* can transstadially transmit ASFV and maintain it for 106 days or more (**Table 1**) (14).

**Table 1:** Summary of published transmission experiments exposing eight *Ornithodoros* species to ASFV.

*Ornithodoros*species	Viralpersistence	Viralreplication	Transovarialtransmission	Transstadialtransmission	Sexualtransmission	ASFVstrains	ASFVinducedmortality	References
*O. marocanus*	655 days	No	–	Yes	–	–	Yes	(19)
*O. puertoricensis*	239 days	Yes	Yes	Yes	–	DR2	Yes	(16, 17)
*O. coriaceus*	502 days	–	No	Yes	–	Tengani; Z1; DR2	Yes	(15, 16)
*O. moubata porcinus*	15 months	Yes	Yes	Yes	Yes	Uganda Tengani; Z1; Ch1 Pr4; Cr1; No6	Yes	(8, 10, 16, 47)
*O. erraticus*	588 days	Yes	–	Yes	Yes	Tomar 87; OUR T88/1 ASFV/P99	–	(43, 48)
*O. moubata* complex	8 months	Yes	–	Yes	–	VIC T90/1; SIN T90/1 CHZ T90/1; LIV 13/33 Georgia2007/1; Ukr12/Zapo	Yes	(37, 49–51)
*O. turicata*	23 days	–	–	–	–	DR2	–	(16)
*O. savignyi*	106 days	No	No	Yes	–	–	–	(14)

## The dynamics of ASFV-infected tick

ASFV can continuously infect natural hosts and domestic pigs, which is of great significance in epidemiological studies. As an important transmission medium of ASF, the study of ASFV infection dynamics in ticks has attracted many researchers’ interests. Here, *O. porcinus* was taken as an example to describe the dynamics after ASFV infection.

ASFV infection happened when *O. porcinus porcinus* feed on blood of viremic pigs. After infection, the midgut was the first site of ASFV replication as determined by Greig, although there were immunofluorescence and virus titration detection of dissected tissues (52). Although the mechanism has not been reported, it could be speculated that the entry of ASFV into midgut epithelial cells may be related to erythrocyte phagocytosis because intact erythrocytes infected with ASFV were detected in the midgut epithelial cells phagolysosomes. It is important to note that most viremic pigs are related to erythrocytic fractions, and almost all field isolates of ASFV are hemadsorbing (53–57). An entry mechanism unrelated to erythrocyte phagocytosis might exist because non-hemadsorbing ASFV was isolated from *O. porcinus porcinus* and experimental ASFV infection could also occur in feeding on an artificial membrane with cell-free virus preparations (47, 47). Greig demonstrated that after being ingested by ticks, ASFV rapidly crosses the gut wall and infect the hemocoel, coxal sac, rectal ampulla, and salivary gland, together with ASFV recovered from the hemocoel and other tissues within 24–48 h of infection, suggesting that there was no true gut barrier to ASFV (52). However, Kleiboeker’s study showed a delay of 15–21 days for ticks to reach generalization of ASFV infection, suggesting the presence of midgut barrier (47).

After transmission and infection in multiple different tissues, the ASFV titer remained at 6 log_10_ HAD_50_/mg of body weight for 290 days of sampling period, which is closely related to that in naturally infected ticks (34, 40, 47). ASFV titers of the reproductive tissue and salivary glands rose to 5–6 log_10_ HAD_50_/mg after 91 dpi, which was the highest of any tissues (47).

Successful ASFV transmission from infected *O. porcinus porcinus* to pigs was associated with high titers in salivary and coxal glands and excretion virus into secretions (8). Surprisingly, coxal secretions were more ASFV positive and had higher ASFV titer than salivary secretions, showing that they were also an important source of ASFV transmission (47). Regurgitation was unlikely to be an effective transmission mechanism for ASFV because the esophageal terminus of argasid species had a proventricular valve, although the tick midgut had high titers of ASFV (58).

## Tick-induced immune suppression

The skin represents the interface where ticks bite and be a site of tick-borne pathogen transmission. Saliva or salivary glands (SG) play a key role in the transmission of most tick-borne pathogens. Tick saliva is a critical biological cocktail that inhibits host defenses and promotes blood flow, containing a large number of molecules involved in cytolytic, anti-coagulant, anti-inflammatory, anti-chemokine, anti-pain, and vasodilating activities (59).

Tick saliva has the ability to suppress innate immune response, complement system, and adaptive immunity of host. In terms of innate immunity, tick saliva strongly suppresses the recruitment of blood-borne innate immune cells. Evasin-1, evasin-2, and evasin-3 are chemokine-binding proteins from *Rhipicephalus sanguineus* SG, which present selectively for different chemokines (60, 61). Ir-LBP, a lipocalin from *I. ricinus*, interfered with neutrophil chemotaxi and activation (62). The saliva of several tick species also contains macrophage migration inhibitory factor (MIF), which inhibit migration of macrophages (63, 64). Tick saliva also inhibits inflammation by reducing or enhancing pro- or anti-inflammatory cytokines secretion, respectively. Hyalomin-A and hyalomin-B exerted significant anti-inflammatory functions by inhibiting the secretion of C–C motif chemokine ligand 2 (CCL2), tumor necrosis factor alpha (TNF-α), and interferon gamma (IFN-γ) and increasing the secretion of interleukin (IL)-10 (65). PGE2 and purine nucleoside adenosine (Ado) also impaired the production of IL-12p40 and TNF-α and increased IL-10 level by murine DC (66).

The complement system links host innate and adaptive immune responses. IRAC I and II from *I. ricinus* inhibited C3 convertase formation of the alternative pathway through blocking complement factor B binding to complement C3b (67, 68). *Ornithodoros moubata* OmCI, TSGP2, and TSGP3 specifically target C5 activation (69, 70). Salp14 and Salp9Pac from *I. scapularis* inhibited mannan-binding lectin binding to the polysaccharide mannan, preventing complement pathway activation (71).

Tick saliva also suppressed the adaptive immunity, including cellular immune response and humoral immune response (72). Iripin-3 from *I. ricinus* reduced IL-6 production by MPs and reduced T-helper type 1 immune response (73). Sialostatin L (SialoL) from *I. scapularis* reduced IFN-γ and IL-17 production and impaired specific T-cell proliferation (74). Salp14 also inhibits pro-inflammatory cytokine production and impaired T-cell proliferation (75). Tick salivary components inhibited humoral immunity by interfering with B-cell-derived immune responses; for example, several ticks could secrete a series of IgG-binding proteins (IGBPs) to suppress specific antibodies (76).

In addition to suppressing immune responses, tick saliva has the ability to block hemostasis and mitigate itching and pain.

## The development of anti-tick vaccine

ASF causes major economic losses with mortality rates approaching 100% and limiting pork production. In addition to anti-viral vaccines, anti-tick vaccine is theoretically an alternative to prevent ASF. In the last decade of the twentieth century, two commercial anti-tick vaccines (TickGARD^©^ and GAVAC^©^) against *Rhipicephalus microplus* were developed based on the glycoprotein antigen Bm86. Regarding soft ticks, antigens that can be used as candidate vaccines are being developed to prevent tick-borne diseases (77).

To date, vaccine development for soft ticks has been significantly less studied than that for hard ticks, and only a few *Ornithodoros* and *Argas* genera species have been involved. Here, the protective effects of candidate antigen of eight soft ticks associated with ASFV transmission are summarized, which include salivary and concealed proteins.

### Concealed antigens

Chinzei and Minoura verified that the egg vitellin of soft ticks was the first recorded study toward concealed antigen, which provided a protective response with 50% reduced fecundity of female tick in immunized rabbits (**Table 2**) (78). Manzano-Román confirmed that the membrane protein extracts of *O. erraticus* midgut epithelial cells had a protective efficacy in mice, pigs, and rabbits (**Table 2**) (79). Oe45, a 45-kDa protein, provided a protective effect on vaccinated host; the mechanism is that anti-Oe45 antibodies immobilize the host complement system and activate it on the intestinal membrane (**Table 2**) (79–81).

**Table 2:** Effective vaccines using concealed antigens from *Ornithodoros* species.

Species	Antigen name	Protein identity	Protein type	Protein function	Host	Protection	References
*O. erraticus*	Egg yolk protein	Vitellin	Native	Embryonic development	Rabbit	50% reduction in oviposition	(78)
	–	–	Membrane proteins of the midgut epithelial cells	–	Pigs, rabbits, mice	50% reduction in females feeding and fecundity, and 80% mortality in nymphs	(79)
	Oe45	Not determined	Native protein, purified from midgut membranes	–	Pig	21–27% reduction in nymph survival, 35%–42% reduction in female feeding and fecundity	(81)
	rOeSub	*O. erraticus* subolesin	Recombinant	Transcription factor	Rabbit	22% reduction in oviposition	(82)
	rOmSub					24.3% reduction in oviposition	
	OE1	*O. erraticus* subolesin	Synthetic peptides	Transcription factor	Rabbit	49% reduction in fecundity	(83)
	OE2					82% reduction in fecundity	
	OM1	*O. moubata* subolesin				50% reduction in fecundity	
	OM2					17% reduction in fecundity	
	OeAQP	*O. erraticus* midgut transcriptomic and proteomic data	Synthetic peptides	Aquaporins	Rabbit	13.4% reduction in fertility	(87)
	OeSEL			Selenoprotein T		26.4% reduction in feeding, 40.5% reduction in fertility, and 6.7% reduction in survival	
	CHI	*O. erraticus* midgut transcriptomic and proteomic data	Recombinant	Chitinase	Rabbit	30.1% reduction in molting	(88)
	TSPs			Tetraspanins		24.8% reduction in molting, 24.7% reduction in oviposition, and 41.4% reduction in fertility	
	RPP0			Ribosomal protein P0		37.1% reduction in molting, 27.7% reduction in oviposition, and 34.1% reduction in fertility	
	PK4			Secreted protein		47.4% reduction in molting, 18.4% reduction in oviposition, and 25.2% reduction in fertility	
*O. moubata*	rOeSub	*O. erraticus* subolesin	Recombinant	Transcription factor	Rabbit	8.5% reduction in oviposition	(82)
	rOmSub	*O. moubata* subolesin				5.2% reduction in oviposition	
	OE1	*O. erraticus* subolesin	Synthetic peptides	Transcription factor	Rabbit	35% reduction in fecundity	(83)
	OE2					40% reduction in fecundity	
	OM1	*O. moubata* subolesin				40% reduction in fecundity	
	OM2					60% reduction in fecundity	
	Om99	*O. moubata* midgut proteome	Recombinant	Protein N-linked glycosylation	Rabbit	Low protection against the *O. moubata* infestations (ranging from 7% to 39%)	(86)
	Om86			Not determined			
	Om85			Not determined			
	Om29			Cell surface receptor signaling pathway			
	Om28			Transmembrane transporter activity			
	Om17			Transmembrane transporter activity			
	Om03			Not determined			
	OeABC	*O. erraticus* midgut transcriptomic and proteomic data	Synthetic peptides	ABC transporter	Rabbit	26.7% reduction in fedding	(87)
	CHI	*O. erraticus* midgut transcriptomic and proteomic data	Recombinant	Chitinase	Rabbit	22.2% reduction in oviposition, and 18.2% reduction in fertility	(88)
	PK4			Secreted protein		17.8% reduction in ingested blood, and 13.3% reduction in molting	

RNA interference with the subolesin gene orthologues had no effect on feeding and survival of *O. moubata* and *O. erraticus* but strongly inhibited tick oviposition, and its recombinant vaccine induced a strong but low protective humoral response (reduced oviposition by 5%–24.5%) in the host (**Table 2**) (82). Then, coupled keyhole limpet hemocyanin (KLH) with four synthetic peptides (OM1, OM2, OE1, and OE2), based on subolesin sequence unrecognized/disordered regions, induced specific antibodies and provided up to 83.1% or 70.1% protective effect in *O. moubata* or *O. erraticus*, respectively (**Table 2**) (83).

Recently, Parasitología et al. sequenced the midgut transcriptomes and proteomes of female *O. moubata* at two physiological conditions, namely, before feeding and 48 h post-feeding, providing a valuable research basis for screening candidate vaccine molecules (84, 85). Next, Prosper screened and recombined five of these candidate proteins (namely, Om17, Om86, OM99, OM85, and OM03), formulated with Freund S adjuvant, and evaluated their immunogenicity and protective effect by vaccinated rabbits. Although these candidate genes had low protective effect against *O. moubata* infection (<39%), they were more effective against *O. erraticus* infection (ranging from 20% to 66%). Two of the five antigens (OM03 and OM85) were considered as an effective anti-tick vaccine and worthy of further study (**Table 2**) (86). Ricardo selected and designed synthetic immunogenic peptides based on the four theoretical candidates, including one selenoprotein T (OeSEL), one ABC transporter (OeABC), and two aquaporins (OeAQP and OeAQP1), which induced humoral responses in vaccinated rabbit, leading to decreased feeding and fertility of tick (**Table 2**) (87). Each of these recombinant proteins for five theoretical candidate antigens, including one chitinase (CHI), one secreted protein PK-4 (PK4), the ribosomal protein P0 (RPP0), and two tetraspanins (TSPs), induced strong humoral responses in vaccinated rabbit, providing a protective effect to *O. erraticus* infestations about 30.2% (CHI), 57.8% (PK4), 57.5% (RPP0), and 56% (TSPs) and cross-protection to *O. moubata* infestations about 19.6% (CHI), 8.1% (PK4), 0% (RPP0), and 11.1% (TSPs). The joint vaccine of these candidates showed a stronger protective effect, with 66.3% protection or 25.6% cross-protection to *O. erraticus* or to *O. moubata*, respectively (**Table 2**) (88).

### Salivary antigens

Astigarraga et al. vaccinated pigs with *O. erraticus* salivary gland extract (SGE), the best effect of which was reduced female feeding and fecundity by 50%. This protective effect is related to three mainly silencing antigens, including proteins of 70, 50, and 20 kDa (89). Subsequently, these proteins were purified to test its protective potential. The pigs produced specific antibodies toward the induced antigens after being vaccinated with either 70- or 50-kDa proteins, thus reducing female feeding and fertility. In contrast, the 20-kDa protein has a poor ability to induce specific antibody responses in pigs (**Table 3**) (79).

**Table 3:** Effective vaccines using salivary antigens from *Ornithodoros* species.

species	Antigen name	Protein identity	Protein type	Protein function	host	Protection	references
*O. erraticus*	70 kDa antigen	–	Native, purified from SGE	–	Pig	Up to 50% reduction in female feeding and fecundity	(79)
	50 kDa antigen	–					
	20 kDa antigen	–					
*O. moubata*	OmC2	Cystatin	Recombinant	Peptidase inhibitor	C3H/HeN mouse	39% reduction in nymph-1 feeding, 15% increase in nymph-1 mortality	(90)
	Om44	–	Native	P-selectin antagonist	Pig	54% reduction in tick feeding, 50% reduction in female fecundity	(91)
	rOmENO	Enolase	Recombinant	Glycolytic enzyme	Rabbit	18% reduction in female fecundity, 20% increase in nymphal mortality	(92)
	PLA2		Recombinant	Secreted phospholipaseA2	Rabbit	23.3% reduction in oviposition, 22.6% reduction in fertility, and 26.6 increase in female mortality	(93)
	APY			Apyrase		26.6% reduction in oviposition, 25.8% reduction in fertility, and 11.7 increase in male mortality	
	MOU			Platelet aggregation inhibitor peptide		31.9% reduction in oviposition, 38.2% reduction in fertility	
	RP-60S	Riboprotein 60S L10				13.2% reduction in oviposition, 10.6% increase in female mortality	
	7DB-like	7DB-like protein				10.7% reduction in fertility	
	rOmTSGP4		Recombinant	Cysteinyl leukotrienes scavenger	Rabbit	16.5 increase in nymph mortality	(94)

Regarding the vaccines against *O. moubata*, Salat et al. showed 39% reduction in feeding and 15% increase in mortality after nymphs-1 took blood meal of recombinant OmC2 cystatin vaccinated C3H/HeN mice (**Table 3**) (90). Similarly, SGE induced various homogeneous protective responses in vaccinated pigs, of which a protein of 44 kDa (named Om44) was the key antigen (89). Purified Om44 vaccinated pigs and rabbits inhibited the feeding of *O. moubata* by up to 54% (**Table 3**) (91). Díaz found that recombinant enolase (rOMENO) vaccinated rabbits caused 18.1% reduction in female oviposition and a rising mortality rates for nymphs-4 and nymphs-3 (**Table 3**) (92), and recombinant proteins of a secreted phospholipaseA2 (PLA2), an apyrase (APY), a mougrin (MOU), a riboprotein 60S L10 (RP-60S), and a 7DB-like protein (7DB-like) induced strong humoral responses, providing protective efficacy of 44.2%, 43.2%, 27.2%, 19.9%, and 17.3%, respectively (**Table 3**) (93). Manzano et al. showed that the recombinant protein TSGP4 (present in the saliva of *O. moubata*) in Freund’s adjuvants provided a 14.1% protective efficacy by activating humoral immunity in a vaccinated host (**Table 3**) (94).

## Discussion

Current strategies to control ASF depend on rapid testing for the virus and the policies of quarantine and slaughter, which result in large numbers of animals being culled and are ineffective. Therefore, prevention is the best way to protect pigs against ASF. Strengthening feeding management, avoiding contact with ASFV-infected pigs, and strictly controlling the quality of pig feed are effective ways to control ASF (95).

In areas where ticks are involved, either in a domestic or sylvatic cycle, tick prevention is also an important part of ASF prevention. Using chemical acaricides is the most basic method of tick control, which has serious shortcomings, including pollution of the environment, contamination of animal products, and the emergence of drug-resistant tick strains (96–98). In addition, there is no guarantee that acaricides will completely kill hidden ticks because they have a nidicolous/endophilic lifestyle, making the use of this strategy inefficient (89). The high cost of developing and commercializing acaricides has prompted the search for alternative methods to control tick.

Ticks are vectors of many zoonotic pathogens, including viruses, parasites, and bacteria, making them a major threat to human and animal health. Alarmingly, more tick-borne diseases are being discovered, such as Alongshan virus and Songling virus (99, 100). Therefore, immunization control with anti-tick vaccines is a promising strategy to prevent tick-borne diseases. Bm86 antigen was identified in *Boophilus microplus* and used as a commercial vaccine against the same tick species. Studies showed that BM86 can effectively inhibit the weight and egg-laying capacity of female adult ticks and total weight of nymphs. Calves immunized with the BM86 vaccine have an ability to resist *B. microplus*, *Boophilus decoloratus*, and *Hyalomma dromedarii (* 101). In addition to damaging the life cycle of ticks, it needs to be evaluated whether anti-tick vaccines block pathogens transmission. Malan showed that *Rhipicephalus appendiculatus* cement protein (64TRP) protected mice against the lethal challenge by infected ticks, which had a protective effect comparable to commercial TBEV vaccine and a better effect in transmission blocking (102). Andaleeb and his colleagues demonstrated that *I. scapularis* salivate gland protein (19ISP) mRNA vaccine inhibited the blood feeding of *I. scapularis* and impeded *Borrelia burgdorferi* transmission from *I. scapularis* to immunized guinea pigs (103). These two cases fully demonstrate the advantages of anti-tick vaccines. The hope for a vaccine against ticks is that it will both reduce tick bites, affect the life stages of ticks after they feed on blood, and reduce pathogens transmission.

The current challenge of an anti-tick vaccine against ASFV transmission is that no candidate anti-tick vaccine has been reported to inhibit transmission of ASFV from ticks to pigs. All the successful anti-tick vaccines described above are used for hard ticks, which differ greatly from soft ticks in their blood feeding habits. Hard ticks attach to hosts for blood meals and last up to 10 days for adulthood (24). Unlike hard ticks, soft ticks feed for a short time; for example, *O. porcinus porcinus* ticks feed in 1 h or less (47). Therefore, it is uncertain whether anti-tick vaccines can prevent ASFV transmission from ticks to pigs. It takes the nymphs 36 h to transmit *B. burgdorferi* to the naive host after feeding on the host, whereas guinea pigs immunized with the 19ISP mRNA vaccine reported by Andaleeb elicited an immune response as early as 18 h after tick challenge, thus blocking *B. burgdorferi* transmission (103, 104). The transmission of the virus is usually within a few hours, so it is necessary to confirm whether the immunized animal can induce an effective immune response to prevent the blood feeding of soft ticks. The effectiveness of the candidate vaccine reported by Ricardo et al. in inhibiting tick blood feeding is limited, so the ability of the vaccine against ASFV transmission is also questionable, which is a problem with all anti-soft tick candidate vaccines reported so far. On the other hand, vaccines against soft ticks have been reported to be effective in the reduction in survival and female fecundity, which is beneficial in reducing tick populations and the risk of ASF and other tick-borne diseases. Interestingly, Jennifer et al. demonstrated that pigs co-inoculated with ASFV and SGE of *O. porcinus* presented increased fever, and SGE had corresponding regulatory effects on skin tissue lesions, Langerhans cells disappearance in epidermis, macrophages recruitment in dermis, and virus dissemination in ASFV infection, suggesting the important role of SGE in the transmission of ASFV (105). Therefore, we are very optimistic about the screening of soft tick antigen and the development of anti-tick vaccine.

Anti-ASFV vaccine is also one of the effective means of prevention and control of ASF. ASFV-G-ΔI177L, marked at 3 June 2022, has become the first commercially available anti-ASFV vaccine due to its protective effect and good safety against Georgia strain. Manuel et al. discovered that ASFV-G-ΔI177L was clinically asymptomatic during the 28-day observational period and exhibited an efficacious protection against the epidemiologically relevant ASFV Georgia isolate (106). Interestingly, Oronasal administration of ASFV-G-ΔI177L provides a protective effect similar to intramuscular administration through mediating ASFV-specific antibody response, such as IgG1, IgG2, and IgM (107). Surprisingly, ASFV-G-ΔI177L also protected pigs from the isolated virulent ASFV circulated and produced in Vietnam (108). Fortunately, virulence regression studies of domestic pigs and large-scale tests of virus shedding and transmission confirmed that ASFV-G-ΔI177L is a safe vaccine (109).

As for the anti-ASFV vaccine, it is undoubtedly the best way to prevent ASF. However, marketed vaccines must be safe without the risk of regaining virulence, which is unpredictable with attenuated vaccines. Since ASFV-G-ΔI177L is a proven safe and effective vaccine, its marketing is good news for pig producers around the world. As there are many serotypes of ASF, the broad spectrum of ASFV-G-ΔI177L needs to be verified. In addition, the duration after vaccination also needs to be verified. Thus, although there is one vaccine available, the development of vaccines for different serotypes in different regions needs to be carried out.

In conclusion, given the critical role of soft ticks in ASFV transmission, the development of both anti-tick and anti-ASFV vaccines is an important strategy for preventing ASF.

## Author contributions

YC, WZ and TL conceived and designed the study. TL, XX, NS, SZ, YD, KL, LD, XC and SJ wrote the manuscript. All authors contributed to the article and approved the submitted version.
